# Comparative efficacy of stereotactic body radiotherapy and percutaneous radiofrequency ablation for oligometastatic liver disease—radiation oncologists’ perspective and practice guideline

**DOI:** 10.1093/bjr/tqag147

**Published:** 2026-06-10

**Authors:** Banu Atalar, Ceren Atahan, Michael Lock, Leandro Cardarelli Leite, Anand Mahadevan, Gamze Ugurluer, Enis Ozyar

**Affiliations:** Department of Radiation Oncology, Acibadem MAA University School of Medicine, Istanbul, 34457, Türkiye; Department of Radiation Oncology, Acibadem MAA University School of Medicine, Istanbul, 34457, Türkiye; Department of Radiation Oncology, University of Western Ontario, London Regional Cancer Program, London, ON N6A 3K7, Canada; London Health Sciences Centre, Schulich School of Medicine and Dentistry, University of Western Ontario, London, ON, N6A 3K7, Canada; Department of Radiation Oncology, NYU Langone Health, New York City, NY, 10016, United States; Department of Radiation Oncology, Acibadem MAA University School of Medicine, Istanbul, 34457, Türkiye; Department of Radiation Oncology, Acibadem MAA University School of Medicine, Istanbul, 34457, Türkiye

**Keywords:** liver, oligometastatic disease, radiofrequency ablation, stereotactic body radiotherapy, guideline

## Abstract

Localized treatment of oligometastatic liver disease can improve both local control and survival. The liver is a frequent site of metastases from colorectal, breast, and lung cancers, but most patients are not eligible for surgical resection due to lesion number, location, or comorbidities. For these patients, non-surgical ablative methods such as radiofrequency ablation (RFA) and stereotactic body radiotherapy (SBRT) are widely used. While RFA has been the traditional approach, SBRT is emerging as a promising alternative, offering precise, non-invasive treatment. SBRT may be especially useful for larger lesions or tumors in locations where RFA is difficult to perform. However, high-quality evidence and large-scale trials are still needed to confirm its efficacy and define its role. This review compares the strengths and limitations of both methods and provides practical guidance for clinical decision-making in the treatment of patients with inoperable liver metastases.

## Introduction

The eradication of metastatic lesions through localized treatments in the oligometastatic setting can improve both local control (LC) and survival.^1^ Liver is among the most common location of oligometastatic disease (OMD), which primarily originate from colorectal, breast and lung cancers and surgical resection of isolated liver metastases has been associated with improved prognosis.^2^ However, 80%-90% of patients with liver metastases are not candidates for surgical resection due to location and number of metastases, inadequate hepatic reserve, or other comorbidities. Therefore, for liver lesions ineligible for surgery, several local and regional ablative methods have been investigated including hepatic artery infusion chemotherapy, transarterial chemoembolization (TACE)/radioembolization (TARE), percutaneous/laparoscopic radiofrequency ablation (RFA) or microwave ablation (MWA), irreversible electroporation (IRE), histotripsy and stereotactic body radiation therapy (SBRT).^3–8^ Ongoing trials are currently comparing various local therapies for the management of liver metastases to identify the most effective treatment options.^7^^,^^9^ The COLLISION trial, the only randomized study comparing ablation and surgery, demonstrated oncological equivalence with a superior safety profile.^10^ These findings suggest that restricting thermal ablation to unresectable colorectal liver metastases should be reconsidered, and treatment should be individualized based on patient and disease characteristics. To date, however, no randomized trial directly comparing SBRT with surgical resection exists and there is no consensus or high-level evidence available of comparative efficacy to establish the optimal approach.

For patients with inoperable liver metastases, traditionally, RFA has been the most utilized treatment method, but it is typically reserved for relatively smaller (<3 cm) metastases that are not in unfavorable locations (eg, subcapsular, subphrenic, or perivascular sites).^11^ Over time, SBRT has gained popularity in managing liver metastases due to its ability to minimize radiation exposure to healthy liver tissue with outcomes comparable to RFA.^2^^,^^12^ As of today, RFA, MWA, and SBRT are among the most used and validated non-surgical approaches, each offering distinct advantages and challenges depending on lesion and patient-specific characteristics. Recently, ASCO guidelines suggested that SBRT may be recommended for CRC patients with oligometastases in the liver who are not considered for resection,^13^ whereas ESMO guideline offers both SBRT and thermal ablative methods, including RFA.^14^ Because majority of the existing comparative oncologic literature has historically used RFA as the reference ablative modality, it was selected as the comparator in this review despite the increasing contemporary use of MWA. Therefore, we aimed to make a comprehensive comparison of the SBRT and RFA as local ablative therapy methods for inoperable liver metastasis and draw a road map to assist clinicians in decision making with a proposed treatment paradigm serving as a practical guide.

## SBRT for liver metastases

Historically, radiotherapy (RT) in liver tumors was limited to palliation where even single fraction RT to whole liver seemed to relieve symptoms in most patients.^15^ But today, with the recent advances in SBRT techniques (fusion of diagnostic images such as PET/CT or MRI with planning CT), image-guidance with gantry-based, robotic-arm or MRI-based linear accelerators (LINAC); motion management including 4D CT or techniques such as breath-hold and tracking; and treatment planning algorithms allowing for the delivery of higher radiation doses in single or multifractions using high dose rates in a short time, SBRT became one of the most convenient, non-invasive and effective ablative treatment for liver metastases. However, its application in the liver is challenging due to the normal liver’s relative radiosensitivity, proximity to radiosensitive organs at risk (OAR) as well as the liver’s motion with respiration.^16^ Several retrospective and prospective studies have reported effectiveness of SBRT for liver metastasis with LC rates comparable to those of surgery and other ablative techniques.^2^^,^^6^^,^^12^^,^^16–31^ Two-year LC and OS range between 30% and 100% and from 30% to 80%, respectively for patients with liver oligometastases.^23^^,^^24^ International Stereotactic Radiosurgery Society (ISRS) meta-analysis including 33 studies with a total of 3101 patients and 4437 liver metastases reported LC rates at 1, 2, and 3 years of 85%, 75%, and 68% respectively, while overall survival (OS) rates were 79%, 54%, and 37%, respectively.^17^ Grade 3 or higher toxicity occurs in less than 10% of cases in all series and less than 5% of cases in more recent series where newer SBRT techniques are available.^12^^,^^16^^,^^23^^,^^32–34^

One of the early pioneering studies from Stanford University in 2011,^35^ reviewed 102 liver metastases treated with a median SBRT dose of 42 Gy in 6 fractions. They observed 1- and 2-year LC rates of 67% and 55%, respectively with no severe toxicity. A significant finding was that LC increased with a biologically effective dose (BED) greater than 75 Gy. A notable trial from Denmark^36^ reported the results of 212 liver metastases in 321 patients with lung and liver oligometastases treated with SBRT. The median metastasis size was 3 cm, ranging up to 9 cm. The study identified that good performance status, solitary metastasis, metastasis less than 3 cm, metachronous metastases, and pre-SBRT chemotherapy were associated with favorable OS. Only one case of grade 5 liver dysfunction and one case of grade 5 late gastritis/perforation were reported. These findings indicated very reasonable toxicity levels, even though older SBRT techniques were used.

Several large multicenter studies have reported the outcomes of patients treated with SBRT for liver metastases. German Radiation Oncology Society (DEGRO),^31^ reviewed 474 patients with 623 liver metastases with predominantly CRC primary, followed by lung, breast, and pancreatic cancers. OS rates were 70% at 1 year and 30% at 3 years. Pre-SBRT chemotherapy and CRC histology were found to be negative predictive factors for metastasis control, likely due to the resistant nature of heavily pretreated CRC metastases. The results indicated that a BED value greater than 150 Gy at the isocenter could achieve LC rates exceeding 80% at 2 years. Additionally, patients treated after 2003 had significantly better LC rates compared to those treated earlier, attributed to the increased use of advanced techniques. Radiosurgery Society Registry trial by Mahadevan et al,^37^ included 427 patients from 25 centers, predominantly featuring liver metastases from CRC, followed by lung and breast cancers. Notably, the median lesion volume was relatively large at 40 cc, and the median dose administered was 45 Gy in 3 fractions. Both smaller lesions and higher BED were associated with improved LC and OS. Specifically, SBRT demonstrated greater efficacy for lesions smaller than 40 cc and treated with a BED greater than 100 Gy. A detailed analysis of the relationship between lesion volume and dose revealed that the significance of a high BED on LC and OS was diminished for lesions less than 4 cm. However, for larger lesions exceeding 40 cc, a higher dose was necessary to achieve superior outcomes. Jo et al^19^ also reported that 2-y LC rates of 52% for BED ≤80 Gy versus 83% for BED 100-112 Gy versus 89% for BED ≥ 132 Gy in their cohort of 70 patients with 103 CRC metastases treated with SBRT. The findings emphasize the necessity of higher BED for the effective treatment of larger lesions.

Recommended doses vary across publications, depending on factors such as the number of the lesions, volume of the lesions, healthy liver volume, and doses to the critical OARs like kidneys, stomach, bowel, spinal cord, bile ducts, major vessels, chest wall and heart. Primary tumor histology and previous treatments should also be considered. Colorectal liver metastasis (CRLM) appears to be more radioresistant compared to lung, breast, and gynecological primaries, but more radiosensitive than melanoma and renal cell carcinoma.^2^^,^^38–40^ In a recent Italian trial, CRC, breast, and lung primary tumors were found to be associated with better outcomes.^41^ However, in the Radiosurgery Society (RSS) study, higher BED_10_ (≥100 Gy) was associated with significantly improved outcomes, regardless of tumor histology.^37^ In the context of prior chemotherapy, the potential for the persistence of radioresistant clones and the risk of missing the subclinical disease in the planning treatment volume (PTV) due to tumor shrinkage should be carefully considered.^39^ Published doses range from 14 to 70 Gy administered in 1-12 fractions. A study conducted in 2016, single-fraction doses of 35 Gy and 40 Gy for non-central liver metastases (outside of 2 cm around the portal vein) were shown to achieve 100% 2.5 y-LC with no severe toxicity.^24^ For lesions near critical organs fractionated schemes with lower doses per fraction or simultaneous integrated boost (SIB) technique—aiming for higher doses in gross target volume (GTV) and lower, safer doses near OARs might be considered.^23^ Although there is no established dose-fractionation schema, multiple trials have suggested that a BED greater than 100 Gy improves both local control and survival rates.^24^^,^^25^^,^^37^^,^^42^

## Interventional radiology methods in management of liver metastasis

There are several interventional radiology methods established for the treatment of liver metastases. Percutaneous approaches, including RFA and MWA, induce cell death through coagulation necrosis. TACE and TARE are vascular catheter-based techniques that cause necrosis via embolization combined with the administration of high-dose chemotherapy or radioactive microspheres, respectively. IRE is a minimally invasive, image-guided treatment for tumors unsuitable for surgery or thermal ablation due to proximity to vital structures such as vessels or bile ducts. It delivers short electrical pulses that disrupt cell membranes by creating nanopores, leading to cell death through loss of homeostasis. In clinical practice, these local therapies are not mutually exclusive; individual lesions within the same patient may be managed using different modalities depending on anatomical feasibility and prior treatment history. Interventional oncology aims to achieve maximal ablation while preserving uninvolved hepatic parenchyma, and repeated or sequential use of these approaches is feasible within a multidisciplinary strategy.

Among these minimally invasive radiology methods, RFA and MWA are the most common and very well-known methods for the treatment of liver metastases. RFA typically performed percutaneously under image guidance with ultrasound or CT to guide a needlelike probe for insertion of the electrode into the lesion, which then emits RF energy to heat the tissue to at least 60 °C for maximum efficacy and cause coagulation necrosis.^4^ It can also be performed laparoscopically, or during open surgery. It is a well evaluated treatment with a local recurrence rate of 5% for lesions less than 3 cm and 20% for larger lesions. However, it poses a risk of “heat sink effect” for perivascular locations and collateral damage in peribiliary and subcapsular locations.^6^ Blood flow in nearby blood vessels absorbs heat, reduces the temperature and diminishes the ablative effect. A subcapsular or high-risk tumor location is regarded as a relative contraindication for RFA. Additionally, lesions larger than 2-02.5 cm require multiple overlapping ablations to achieve complete necrosis^4^ and tumor control rates drops for tumor sizes larger than 3 cm.^43^ MWA achieves larger areas of cellular destruction, requires less time to perform, delivers higher temperatures, and is less affected by heat-sink effects. However, the expanded zones of necrosis can raise the risk of complications due to potential damage to surrounding healthy tissues. MWA is currently regarded as more effective than RFA for treating perivascular lesions, while RFA is considered safer for peribiliary metastases due to its less intense heat generation and more predictable ablation zones,^4^ however non-thermal ablation techniques such as IRE are generally considered the preferred option for lesions adjacent to major bile ducts. The technical differences between these techniques is beyond the scope of this review.

## Comparison of SBRT vs. RFA for liver metastases

### Overview

To date there are no randomized prospective trials in the literature to compare interventional radiology methods (RFA or MWA) with SBRT. However, there are few retrospective comparisons and metanalysis available. Among these, the Michigan University reported the largest cohort retrospectively,^30^ of 161 patients with 282 metastases, predominantly from CRC. The median SBRT dose administered was 50 Gy, with a notably larger median lesion size treated with SBRT. The study demonstrated a 2-year LC rate of 88% for SBRT compared to 61% for RFA, favoring SBRT (*P* = .06) with no difference in 2-year OS with rates of 51%. Multivariate analysis revealed that treatment with SBRT and smaller metastasis size were associated with improved LC.

In the meta-analysis involving over 2000 patients comparing RFA and SBRT from Korea,^44^ only 3 trials included patients with liver metastases, while the remaining 8 trials focused solely on HCC. The analysis revealed that including both HCCs and liver metastases, SBRT exhibited a higher 2-year LC rate compared to RFA. Specifically, for liver metastases, the pooled 2-year LC rates were 60% for RFA and 83% for SBRT, in spite of favorable smaller tumors receiving RFA, indicating superior outcomes with SBRT. Interestingly, neither this meta-analysis nor other comparative trials found any significant difference in treatment-related toxicity between RFA and SBRT, with both modalities demonstrating reasonably low toxicity levels. However SBRT is relatively more non-invasive than percutaneous approaches with no requirement of anesthesia or analgesia.

Dutch prospective registry comparing thermal ablation (RFA or MWA) with SBRT analyzed a total of 199 patients with 144 of thermal ablation and 55 of SBRT. Thermal ablation demonstrated superior OS (median OS 53.0 months vs. 27.4 months) and LC compared to SBRT, which could be due to selection bias favoring RFA for smaller tumors. Both treatments had low morbidity, with no serious adverse events in the SBRT group and a 6.3% rate of grade 3 adverse events in the thermal ablation group. Metastasis size was the strongest predictor of local tumor progression in the thermal ablation group but has no influence in the SBRT group, indicating that SBRT may be less affected from lesion size.^18^

In another retrospective Korean series comparing 222 colorectal cancer patients with 330 liver metastases treated with RFA vs. SBRT, no significant differences were found in 1-year and 3-year recurrence-free survival (35% vs. 43%, 22% vs. 23%), OS (96% vs. 91%, 58% vs. 56%), and freedom from local progression (FFLP; 90% vs. 72%, 78% vs. 60%). The median lesion size was significantly smaller in the RFA group compared to the SBRT group (1.5 cm vs. 2.3 cm). In lesions larger than 2 cm, the SBRT group had higher FFLP rates. Additionally, more lesions in difficult locations were treated in the SBRT arm (61% vs. 91%). They concluded that lesion size was an independent prognostic factor for LC and SBRT may be preferred for larger lesions.^21^

A meta-analysis conducted in 2023, including over 4600 patients, compared RFA and SBRT in liver malignancies, including HCC and liver metastases. The median 2-year OS rates were 64.3% in the RFA group and 65.4% in the SBRT group, while the median 2-year LC rates were 60.8% in the RFA group and 77.0% in the SBRT group for metastases.^6^ The overview of studies comparing SBRT and RFA in the liver metastasis treatment is summarized in Table 1.

**Table 1:** Studies comparing SBRT and RFA for liver metastases.

Study	Design	Number of patients and lesions	Tumor size	Treatment groups	Key findings
**Jackson et al. (2018)^30^**	Retrospective	161 Patients with 282 liver metastases	Median tumor size: 2.7 cm (SBRT) vs. 1.8 cm (RFA)	SBRT (*n = *170) vs. RFA (*n = *112)	SBRT showed a 2-year FFLP rate of 88.2% vs. 73.9% for RFA (*P = .*06). For tumors ≥2 cm, SBRT had improved FFLP (HR, 0.28; 95% CI: 0.09-0.93). No significant difference in OS between groups.
**Lee et al. (2020)^44^**	Systematic review and meta-analysis	2238 Patients with CRLM and HCC	Not specified	SBRT vs. RFA	SBRT demonstrated significantly higher 1- and 3-year LC rates compared to RFA. However, 2-year OS rates were significantly lower for SBRT than RFA (OR: 0.63; 95% CI: 0.51-0.79; *P < .*0001).
**Nieuwenhuizen et al. (2021)^18^**	Retrospective	199 Patients; 469 CRLM	Median tumor size: 2.9 cm (SBRT) vs. 1.4 cm (Thermal ablation including RFA and MWA)	SBRT (*n = *69) vs. Thermal Ablation (*n = *400)	SBRT was associated with inferior OS (median OS 53.0 months vs. 27.4 months), inferior LTPFS per-tumor (HR = 1.24, 95% CI 1.01-1.52; *P = .*044) and LC per-patient (HR = 1.57, 95% CI 1.20-2.04; *P = .*001) and per-tumor (HR = 1.89, 95% CI 1.44-2.49; *P < .*001)
**Yu et al. (2022)^21^**	Retrospective	222 Patients with 330 CRLM	Median tumor size: 2.3 cm (SBRT) vs. 1.5 cm (RFA)	SBRT (*n = *62) vs. RFA (*n = *238)	Favoring SBRT with tumours > 2 cm (HR 0.153, *P < .*001), no difference in LC with tumours <2 cm (HR 0.648, *P = .*1), LC 58% vs. 76% (RFA vs. SBRT). More tumours in difficult location in SBRT arm (60.7% vs. 90.9%, *P* = .001)
**Rim et al. (2023)^6^**	Meta-analysis	4638 Patients with CRLM and HCC	Not specified	SBRT (*n = *196) vs. RFA (*n = *433) for CRLM	1- and 2-Year survival rates for metastasis studies were 88.2% and 66.4% after RFA and 82.7% and 60.6% after SBRT, respectively. No significant difference in OS between groups.

Abbreviations: CRLM, colorectal liver metastasis; FFLP, freedom from local progression; HCC, hepatocellular carcinoma; LC, local control; LTPFS, local tumor progression-free survival; MWA, microwave ablation; OS, overall survival; RFA, radiofrequency ablation; SBRT, stereotactic body radiotherapy.

### Clinical decision-making

The approach to metastatic liver lesions should involve a multidisciplinary team. Only patients with sufficient liver function should be eligible for local ablative treatments (Table 2). Patients with more than 5 liver metastases, metastases larger than 6 cm in diameter, abutment or <5 mm to gastrointestinal structures, inadequate liver function (Child-Pugh C), or a free liver volume <700 cm³ are generally not advised to undergo SBRT.^45^ Clinicians should also be aware that post-treatment evaluation differs markedly between thermal ablation and SBRT. After RFA, immediate contrast-enhanced ultrasound or CT can confirm complete ablation, and repeat treatment is possible if residual enhancement is seen. In contrast, SBRT response assessment requires a longer interval, as transient post-radiation enhancement and edema may persist for several weeks. CT is less reliable for early evaluation, whereas MRI and PET/CT are preferred for late response assessment.^4^^,^^42^

**Table 2:** Patients eligible for liver SBRT and RFA.

Criteria
ECOG status 0-2 for SBRT and 0-1 for RFA
Adequate liver function: bilirubin <3 mg/dL, albumin >2.5 g/dL, tumor-free liver volume >700 cm³ for SBRT
Adequate hematological function: absolute neutrophil count (ANC) >1.5 × 10^9^/L. Platelets >100 × 10^9^/L. Hemoglobin (Hb) >9 g/dL
Normal PT (>70%) and PTT, except in patients using anticoagulants
Liver enzymes <3× the upper limit of normal
Adequate renal function for the infusion of intravenous contrast agents

### Factors to consider selecting the local ablative methods

#### Patients’ performance status

Liver SBRT is a primarily noninvasive treatment, making it suitable for patients with lower performance status (ECOG 0 to ECOG 2) or those who cannot tolerate invasive procedures. It generally does not require anesthesia, and the lack of physical intervention minimizes the risk of mechanical complications or infections. However, in some cases, fiducials may be implanted to improve targeting precision. While SBRT does not typically require hospitalization and allows patients to quickly return to their daily routines, it still demands patient compliance. Patients must tolerate the supine position for extended periods and adhere to advanced techniques like breath-hold methods, MR-guided RT, and longer treatment sessions. RFA, on the other hand, is minimally invasive, requiring the insertion of a needle or probe into the tumor, which necessitates intravenous sedation or general anesthesia with standard cardiac, pressure, and oxygen monitoring. This can limit its use in patients with poor performance status or comorbidities that increase anesthesia risks. Like SBRT, RFA is typically performed as an outpatient procedure, but it carries a slightly higher risk of complications such as intraperitoneal hemorrhage, portal vein thrombosis, biloma, and bile duct injury.^3^

#### Location of metastasis in the liver

SBRT has no limitations in terms of metastasis location in the liver, making it ideal for treating lesions near critical structures such as major blood vessels or bile ducts, where it can deliver high-precision radiation without damaging these surrounding structures. RFA is more suited for superficial, easily accessible lesions but faces challenges when treating those near major blood vessels, adjacent to the biliary tract or gallbladder, or beneath the diaphragm. RFA’s effectiveness decreases near major vessels due to the heat-sink effect, where the cooling blood flow reduces the ablation’s efficacy. RFA in metastases adjacent to the hepatic hilum increases the risk of injury to the biliary tract, and in those adjacent to the gallbladder hilum increases the risk of iatrogenic cholecystitis.^46^ Hydrodissection use may help mitigate the risk.

Moreover, RFA has historically relied on tumor visibility with ultrasonography, which may be problematic for less visible tumors. Most centers now use CT for margin assessment and there is now emerging evidence for the use of CT with hepatic arteriography with direct injection of contrast to the hepatic artery making the visualization of the tumors much better. SBRT, however, does not depend on ultrasound visibility but rather on CT- or MRI-based target delineation. However, in patients where fiducial marker placement is needed for SBRT, collaboration with interventional radiology is essential. Sometimes small changes are difficult to visualize on MRI and CT scans during RT planning, and respiratory artifacts may further compromise image quality. Advances in image fusion techniques, which combine CT with MRI or PET imaging, have improved tumor localization for SBRT. The use of MR-guided or fiducial-free image guidance platforms^16^ enable the delivery of high-dose radiation to tumors that are in high-risk areas for RFA. As a result, SBRT has shown better LC in treating subphrenic and segment 8 tumors in HCC patients.^47^

### Size and number of metastases

SBRT mostly is not affected by lesion size, maintaining good LC and toxicity outcomes regardless of lesion dimensions. However, visualization of small subcentimeter lesions, which may be only visible as foci of diffusion restriction on MRI, may pose a practical challenge. The integration of MRI—including diffusion-weighted and hepatobiliary phase sequences—into planning CT for precise target contouring is essential. Scorsetti et al^2^ found no difference in LC between lesions larger than or smaller than 3 cm when treated with SBRT. However, a PTV over 50 mL was associated with poorer overall survival, while a PTV under 104 mL was linked to better outcomes in different trials.^48^^,^^49^ Several trials used a maximum metastasis diameter limit of 6 cm as inclusion criteria.^12^^,^^43^^,^^50–52^ However, there are also trials with largest lesion >6 cm in diameter.^48^^,^^53^ Lesion size ≥2 cm was found to be associated with worse LC after RFA, but not after SBRT.^21^^,^^30^ When independently analyzing patients treated with RFA and SBRT, increasing lesion size was predictive of decreased LC for patients treated with RFA, with nearly a 2-fold risk of local progression for each centimeter increase in lesion size.^30^ Machi et al^5^ reported local recurrence rates for RFA of 3%, 15%, and 50% for lesions measuring less than 4 cm, 4-10 cm, and greater than 10 cm, respectively. Therefore, larger lesions may require repeated sessions of RFA and unlike SBRT or surgery, an important advantage of RFA which would be feasible unlike SBRT or surgery, which are limited by liver reserve.

Both in HCC and liver metastases trials, SBRT appears to have an advantage, particularly for lesions larger than 3 cm, while RFA shows a decline in efficacy for lesions larger than 2 cm.^30^^,^^54^^,^^55^ This is a limitation not shared to the same extent by MWA which has similar efficacy up to 3 cm, with ongoing trials in larger lesions.^7^

There is no consensus on the upper limit of the number of metastases for SBRT. It is generally advised to treat up to 3-5 metastases; however, as long as there is sufficient functional liver, some trials have included patients with up to 14 metastases.^56^ Similarly, EORTC 40004 trial included patients up to 10 metastases for RFA but with a limit of 4 cm diameter.^57^ Nevertheless, most of the centers use RFA for treating up to ≤5 metastases.^46^

#### Combination with systemic therapies

RFA, being an invasive procedure, may require a pause in systemic treatments, particularly anti-VEGF, due to the increased risk of bleeding or complications during the procedure. Despite these considerations, RFA has been shown to be safe when combined with chemotherapy.^58^

There is limited data on combining treatments with SBRT and data for liver SBRT is either extrapolated or based on analogies from other tumor sites. Only a few agents, such as crizotinib, erlotinib, immune checkpoint inhibitors, and LHRH analogues, have shown acceptable tolerance when used with SBRT.^25^^,^^59^ The EORTC-ESTRO OligoCare consortium agreed that trastuzumab and pertuzumab can be administered on the same day as SBRT, while other targeted therapies or immunotherapy should be interrupted at least 1 to 2 weeks before and after SBRT.^60^ In cases where data is lacking, considering the drug’s half-life, cessation of systemic treatment at least 7 days before SBRT is recommended according to Australian guidelines.^61^ Importantly, no changes of dose or fractionation were suggested in any of the recommendations.

Concomitant immunotherapy and radiation has been explored due to synergistic abscopal effects. However, apart from anecdotal reports, prospective evidence is lacking.

#### Adverse event

Liver SBRT generally has a favorable adverse event profile, with most patients experiencing mild side effects such as fatigue or temporary liver enzyme elevation. However, several potential adverse events merit attention. These include radiation-induced liver disease (RILD), perforation, bleeding, stenosis, particularly when neighboring the gastric, duodenal, or biliary tract. The risk of serious complications is minimal due to the precision of the radiation delivery. In International Stereotactic Radiosurgery Society (ISRS) meta-analysis, grade 3 and 4 toxicity occurred in 3% of the 3101 patients.^13^ RFA, on the other hand, seems to carry a higher risk of complications with a range of 2%-45%,^4^ due to the invasive nature of the procedure and the vast majority of complications are seen as a result of mechanical damage from the procedures (eg, bleeding, perforation, and pneumothorax).

#### Cost-effectiveness and financial burden to health care system

RFA is generally more cost-effective because it is typically a single-session procedure, making it less expensive in terms of equipment and hospital costs. SBRT involves multiple fractions and advanced imaging technology, motion management, complex treatment device, physics calculations making it more expensive. In a trial where both treatments were assumed to offer similar median survival, comparing the costs of 3 fractions of SBRT and RFA, it was found that SBRT is significantly more expensive. However, SBRT may become a cost-effective option if it demonstrates even a small improvement in survival.^62^ Large-scale clinical trials are needed to establish its role both in comparison to the data for RFA and more modern ablative techniques including MWA.

## Conclusion

Based on current evidence, SBRT may be considered for larger liver metastases (>3 cm) in preference to other ablative techniques when surgery is not feasible. Treatment decisions should be individualized within a multidisciplinary team setting, considering local expertise, technical feasibility, and patient-related factors. SBRT may offer an effective and safe noninvasive treatment option; however, high-quality prospective studies are still needed to further define its efficacy and optimal role in the management of liver metastases.
